# Today Prospects for Tissue Engineering Therapeutic Approach in Dentistry

**DOI:** 10.1155/2014/151252

**Published:** 2014-10-14

**Authors:** Maurizio Bossù, Andrea Pacifici, Daniele Carbone, Gianluca Tenore, Gaetano Ierardo, Luciano Pacifici, Antonella Polimeni

**Affiliations:** Department of Oral and Maxillofacial Sciences, Sapienza University of Rome, Via Caserta 6, 00161 Rome, Italy

## Abstract

In dental practice there is an increasing need for predictable therapeutic protocols able to regenerate tissues that, due to inflammatory or traumatic events, may suffer from loss of their function. One of the topics arising major interest in the research applied to regenerative medicine is represented by tissue engineering and, in particular, by stem cells. The study of stem cells in dentistry over the years has shown an exponential increase in literature. Adult mesenchymal stem cells have recently been isolated and characterized from tooth-related tissues and they might represent, in the near future, a new gold standard in the regeneration of all oral tissues. The aim of our review is to provide an overview on the topic reporting the current knowledge for each class of dental stem cells and to identify their potential clinical applications as therapeutic tool in various branches of dentistry.

## 1. Introduction

Anatomical structures of the mouth undergo several physiologic and pathologic modifications which can determine damages towards both hard and soft tissues [1]. One of the purposes of the scientific research in medical field is to provide techniques and materials to repair the loss of damaged tissues. In recent years a new approach based on tissue engineering is now adding the current treatment protocols. Tissue engineering was introduced in the 1990s and consists of an ensemble of techniques and procedures aimed at the regeneration of biological tissues [2] based on a triad derived from the three major components of tissues: cells, their ECM, and a signalling system [2].

**Figure 1:**
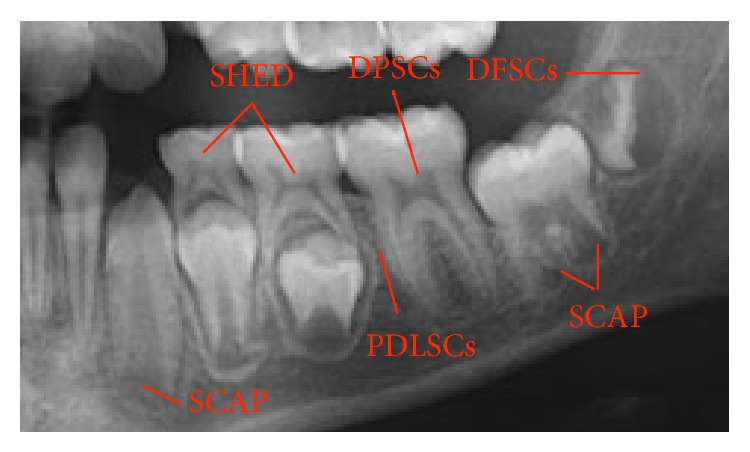
Dental stem cells' sources.

Stem cells are generally defined as clonogenic cells capable of both self-renewal and multilineage differentiation [3] and have been identified from three main sources: embryonic stem cells, adult stem cells, and induced pluripotent stem cells [1]. Embryonic stem (ES) cells are pluripotent cells derived from blastocyst-stage embryos; pluripotent stem cells have not undergone complete differentiation and retain the capacity to divide into any of the three germ layers (endoderm, ectoderm, and mesoderm) but not into extraembryonic tissue [4]. Adult, somatic or postnatal stem cells reside amongst differentiated cells within a number of organs in the body where they play a role in tissue maintenance, renewal, and repair. They are multipotent stem cells and are more restricted in their differentiation capacity when compared with embryonic stem cells [1]. Induced pluripotent stem (iPS) cells are the product of somatic cell reprogramming to an embryonic-like state through genetic manipulation [5]. They have been first developed from adult mouse cells and then from adult human cells [5, 6]. Several types of adult stem cells have been isolated from teeth (Figure 1), including dental stem cells (DPSCs) [7], periodontal ligament stem cells (PDLSCs) [8], stem cells from human exfoliated deciduous teeth (SHEDs) [9], dental follicle progenitor stem cells (DFPCs) [10], and stem cells from apical papilla (SCAPs) [11].

In order to use stem cells in tissue engineering procedures, the presence of a scaffold and growth factors is necessary [2]. An ideal scaffold should support the attachment, migration, proliferation, and spatial organization of cells required for structural and functional replacement of the target tissue [12]. Growth factors (GFs) are peptide molecules which transmit signals to control cell behavior and activity interacting with specific receptors located on the surfaces of cells [13].

**Figure 2:**
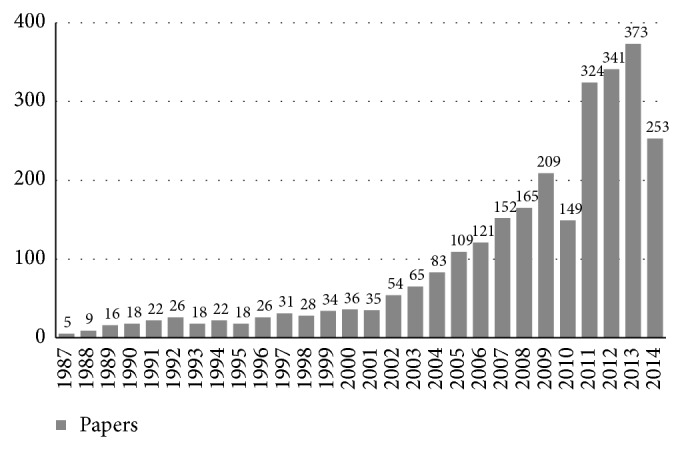
Number of papers dealing with stem cells in dentistry published during the last 27 years.

In recent years there has been an exponential increase in the number of publications dealing with stem cells (Figure 2). The focus of stem cells research in dentistry is the regeneration of missing oral tissues like, for example, dentine-pulp complex, maxillary bone, and periodontal ligament [14]. A further interest of dentistry towards stem cells is due to the fact that it is possible to isolate and harvest them from dental tissues; the oral cavity must be considered as a source of stem cells as well as a site of application.

The aim of our review of literature was to define various dental stem cells types and outline their possible modalities of clinical application for tissue regeneration.

## 2. Material and Methods

In order to perform our revision we consulted PUBMED database initially performing several test searches. Afterwards we decided to carry out the ultimate search by entering “STEM CELL” as main inquiry term, “AND” as default Boolean operator, and “IN DENTISTRY” as secondary inquiry term and we added four search filters offered by the same database. Therefore, review articles were excluded by the results even if these have been studied for the purpose of completeness of the research. Then we performed a second exclusion step by reading articles' title and abstract and a third exclusion step by reading original manuscripts. In our work mainly papers focused on in vivo studies on stem cells in tissue engineering applications were considered in order to investigate on the current knowledge about feasible usage of stem cells as regenerative tool for therapeutic purposes.

Our primary search resulted in a total number of 586 articles including 129 revisions on the subject. We considered 116 papers by reading title and abstract and we finally included 40 papers by analyzing the complete manuscript content.

## 3. Results and Discussion

Data extracted from the analysis of the selected articles are summarized in Table 1.

### 3.1. DPSCs

Adult human dental stem cells were first identified and isolated in 2000 by Gronthos et al. [7] from normal impacted third molars' pulp and were characterized as clonogenic and highly proliferative, being able to form in vitro calcified sporadic nodules [7].

DPSCs are shown to anatomically locate in a perivascular niche within the pulp tissue [15] and to possess self-renewal capabilities and multipotent differentiating ability: they can differentiate in vitro into odontoblasts, adipocytes, neural cells, osteoblasts, chondrocytes and myoblast-like cells [16–20]. Interestingly DPSCs have also been reported to show immunomodulatory properties in vitro and in vivo on mouse [21]. Although they share several characteristics with bone marrow mesenchymal stem cells (BMMSCs), DPSCs show reduced osteogenic and adipogenic potentials in vitro when compared to BMMSCs [7].

Human DPSCs can also be successfully isolated and characterized from inflamed pulp tissue [22], from supernumerary teeth [23] and from natal teeth [24]. Moreover several studies have isolated and characterized stem cells and subpopulations of progenitor cells in the dental pulp of different animal species [25–27].

The banking of DPSCs by cryopreservation in liquid N2 is clinically possible for future usage providing a good prospective in future regenerative dental and medical treatment [28, 29]. DPSCs have been successfully isolated from cryopreserved healthy molar and premolar teeth, as well as from their undigested dental pulp tissue [30–33] and also from diseased but vital teeth [34].

DPSCs' possible employment as therapeutic tool in regenerative endodontics is supported by several in vivo studies which showed that human DPSCs transplanted under the skin of immunocompromised mice formed pulp/dentin-like tissue complexes after odontoblastic differentiation [7, 35–37]. Different scaffolds were used in these studies. Gronthos et al. used a hydroxyapatite/tricalcium phosphate (HA/TCP) ceramic powder scaffold [7]. Demarco et al. used Poly-L-lactic acid (PLLA) scaffolds prepared in pulp chambers of extracted human third molars using salt crystals or gelatin spheres as porogen (PLLA/tooth slice scaffold). This study showed that dentin-related morphogen factors influence the differentiation of stem cells toward an odontoblast-like cell phenotype [35]. Prescott et al. and Johnson et al. used a collagen scaffold and Dentin Matrix Protein-1 (DMP1) [36, 37] which is a growth factor that is primarily found in dentin and bone and has been implicated in the regulation of mineralization processes [38]. A very interesting suitable scaffold for regenerative endodontics is a self-assembling peptide hydrogel that can be poured into a pulp chamber and which self-polymerizes under physiological conditions to form a solid gel capable of supporting cell growth and differentiation [39]. Its application is highly attractive from an endodontic stand-point as a liquid may be expected to conform more easily to the variable shape of a pulp chamber than would a solid or even moldable scaffold. Human DPSCs have been reported to generate pulp-dentin-like complex also arranged in 3D scaffoldless structures in human root canals implanted subcutaneously into mice [40].

Animal DPSCs have also been tested in several in vivo experiments for regenerative endodontics [41–46]. DPSCs might have possible application in bone regenerative procedures. Nakamura et al. reported that rat dental pulp cells have the potential to generate mineralized tissue via the osteoblastic phenotype, on titanium, in vitro [47]. Some investigators reported the successful formation of lamellar bone in vivo by inducing human DPSCs to synthesize bone tissue in vitro and then transplanting it subcutaneously into mice, without needing of a scaffold support as the transplanted fibrous bone was an already formed hard tissue [48, 49]. Chan et al. used a self-assembling peptide hydrogel scaffold seeded with DPSC to create mineralised bone-like tissue pieces containing blood capillaries [50]. DPSCs with a mature osteogenic phenotype have been reported to be more responsive to pulsating fluid shear stress than osteogenically immature DPSCs and produce more bone in vivo suggesting that DPSCs with a mature osteogenic phenotype might be preferable for bone tissue engineering, because they might be able to perform mature bone cell-specific functions during bone adaptation to mechanical loading in vivo [51].

Nishino et al. reported a possible application in soft tissue regenerative medicine for human DPSCs associated with Basic Fibroblast Growth Factor (b-FGF), which where shown to accelerate the wound healing of a skin defect of a mice [73]. A study performed by Khorsand et al. showed that Dog DPSCs seeded on bovine bone granules possess periodontium and bone forming ability in periodontal canine defects [56].

### 3.2. PDLSCs

Adult stem cells from human periodontal ligament (PDL) of healthy permanent teeth were first isolated and characterized in 2004 by Seo et al. [8]. PDLSCs possess classic characteristics of stem cells (i.e., small size, slow cellular cycle, and several stem cells markers' expression) [8, 74] and show a faster cell growth and superior clonogenic capabilities compared with BMMSCs [75].

PDLSCs were shown to possess multilineage differentiation ability in vitro into osteoblast-like cells, cementoblast-like cells, adipocytes, and collagen-forming cells [8, 75, 76] although their osteogenic potential was found to be lower than their bone marrow and pulp tissue counterparts in vitro [8, 75]. PDLSCs have been reported to differentiate into chondrocyte-like cells in chondrogenesis-inducing media with the addition of Transforming Growth Factor *β*3 (TGF-*β*3) [75] and by adding TGF-*β*3 and BMP-6 to the culture [77]. They have also been reported to possess immunomodulatory functions which might lead to new possible application fields [78].

PDLSCs have been isolated from inflamed regenerating periodontal tissue obtained from intrabony defects during flap surgery and showed similar proliferating and differentiation properties, an increased migratory capacity, and a lower osteoblastic differentiation ability when compared to healthy PDLSCs [60, 79]. Stem cells isolated from periodontitis-affected periodontal tissue were even shown to differentiate into highly proliferative neural precursors in vitro [80].

Several authors investigated the differences between PDLSCs isolated in permanent or in deciduous teeth; deciduous PDLSCs were found to have a higher proliferative rate [61, 81] and both cell types display multipotentiality toward adipocytes, osteoblasts, and chondrocytes with some differentiation potential differences among them [82]. Silvério et al. reported that deciduous PDLSCs have higher ability to differentiate into adipocyte-like cells, rather than osteoblast-like cells, compared to permanent PDLSCs [81]. Conversely Ji et al. reported that deciduous PDLSCs are more apt than permanent PDLSCs to differentiate into both osteoblasts and adipocytes under appropriate differentiating in vitro conditions [61].

Several studies have isolated and characterized stem cells and subpopulations of progenitor cells in the PDL of different animal species [58, 83, 84].

FGF-2 was found to increase proliferation of the human PDLSCs cultures [85]. Moreover it has been shown that TGF-*β*1 combined with PDGF-BB and IGF-1 stimulated mitogenesis and enhanced the adhesion of human PDL cells to human periodontally diseased root fragments treated by scaling and root conditioning with a citric acid and tetracycline solution [86]. Swine PDLSCs have been reported to be induced by BMP-2 to form mineralized nodules and by FGF-2 to form tube-like vascular structures [84].

Several in vivo studies have been performed on PDLSCs. Human PDLSCs mixed with HA/TCP ceramic particles have been shown to be capable of generating a cementum/PDL-like complex, characterised by a layer of aligned cementum-like tissues and clearly associated PDL-like tissues, when transplanted into periodontal defects surgically created in rats [8]. PDLSCs from both healthy and inflamed human PDL mixed with macroporous biphasic calcium phosphate have been reported to create a typical cementum-like/PDL structure after transplantation into immunocompromised mice. However, the degree of cementum regeneration induced by the inflamed DPSCs was significantly lower than that induced by healthy PDLSCs [60]. Stem cells from human deciduous and permanent PDL have been compared in vivo; deciduous PDLSCs cell sheets combined with dentin blocks transplanted into the peritoneal cavity of nude mice were able to generate regularly arranged PDL-like fibrous tissue that interfaced with new cementum-like tissue formed on the surface of the dentin block. In contrast, there was only PDL-like tissue regeneration, without cementum formation, in transplanted human permanent PDLSC cell sheets [61].

Canine PDLSCs have been reported to generate a cementum/PDL-like complex if seeded on a HA scaffold and transplanted into immunocompromised mice [58] and also if applied into furcation defects on dog using a collagen scaffold [57]. Furthermore PDLSCs from dog have been found to promote bone regeneration mixed with HA/TCP carriers in surgically created peri-implant saddle-like defects [59] and supported by bovine bone granules in sinus floor augmentation on dog [62].

Rat PDLSCs seeded on a gelatin sponge have been referred to promote bone, PDL, and cementum formation in vivo on rat [63].

### 3.3. SHED

SHED represent a distinctive population of multipotent stem cells from the remnant pulp of exfoliated deciduous teeth [9] which derive from a readily accessible tissue source as human deciduous teeth that are expendable and routinely exfoliated in childhood with little or no morbidity to the patient [9, 70, 71].

Although both are extracted from pulp tissue, SHED and DPSCs exhibit significant differences regarding proliferative capacity and gene expression, which can potentially affect their mechanisms of differentiation [87]. SHEDs express mesenchymal stem cell markers such as DPSCs, but exhibit a significantly higher positivity for CD146, a multipotency related marker for mesenchymal stem cells whose expression denotes less differentiated lineages which may have a higher differentiating capacity [64]. SHEDs exhibit a higher proliferation rate than DPSCs in vitro [9, 64] and the capacity to differentiate into several mesenchymal lineages, such as osteoblasts, odontoblasts, adipocytes, chondrocytes, and myocytes and expressed neuroprogenitor markers [9, 64, 88, 89]. Stem cells from deciduous teeth pulp are obtained easier in teeth with advanced resorption process probably because of the modifications in the ECM performed by the high quantities of cytokines produced by circulating mononuclear cells involved in the resorptive phenomenon [90].

In vitro tests showed that SHEDs have a higher capacity than DPSCs for osteogenic and adipogenic differentiation [9, 64].

In vivo studies carried out by implanting tooth slice/PLLA scaffolds containing SHED into the subcutaneous tissue of immunodeficient mice, showed that SHEDs possess the ability to develop a dental pulp-like tissue and vascular structures anastomosed with the mouse vasculature. In this particular study model dentin-derived morphogenic signals are necessary and sufficient to induce the differentiation of stem cells into odontoblasts [69, 70]. Another scaffold model supporting the odontoblastic differentiating ability of SHED consisted of peptide hydrogel or human recombinant collagen (rH collagen) injected into human tooth root and transplanted into mice [66]. The ability of forming pulp-like tissue in vivo was also reported by using a macroporous biphasic calcium phosphate scaffold and fibroblast growth factor-2 (FGF-2) with SHED from inflamed deciduous teeth [68].

The capacity of osteogenesis of SHED was supported by in vivo experiments in which SHED, arranged in cell pellets or mixed with ceramic bovine bone or HA/TCP scaffolds and transplanted into animal models, underwent osteoblastic differentiation and determined bone tissue formation [9, 64, 67, 71]. SHED showed a higher bone forming ability in vivo than DPSCs when transplanted in the same experimental conditions [64]. SHEDs are reported to have significant immunomodulatory properties in vitro and in vivo when transplanted in mice [91].

### 3.4. DFSCs

DFSCs have been isolated and characterized by Morsczeck et al. from normal human impacted third molars [10]. They show a typical fibroblast-like morphology and express mesenchymal stem cell markers [10]. DFSCs were able to differentiate in vitro in PDL-like structures or calcified nodules with bone- or cementum-like attributes [10]. Honda et al. found that DFSCs demonstrate osteogenic-, adipogenic-, and periodontium-like tissues differentiation capacity in vitro after induction but they are not able to differentiate in chondrocytes [53] while Kémoun et al. reported that they can differentiate into osteoblasts, chondrocytes, and adipocytes [92]. DFSCs have also been successfully cultured into a serum-free medium [93].

DFSCs have been tested in vivo in several studies [10, 52–55]. Human DFSCs have been transplanted with HA powder into immunocompromised mice and generated a structure lining the surfaces of the HA particles, which are comprised of fibrous or rigid tissue. In this study no cementum or bone formation was found in histological sections [10]. Pellets of human dental follicle cells have been reported to be able to regenerate critical size bone defects in rats' calvaria [53]. Human DFSCs cells mixed with porous ceramic discs showed hard tissue-forming potential in immunocompromised rats [55]. Porcine DFSCs mixed with *β*-TCP formed mineralized bone-like tissue subcutaneously in immunodeficient mice [54]. Bovine DFSCs mixed with HA powder and transplanted into immunocompromised mice generated cementum-like mineralized tissue on the border of HA beads and a ligament-like fibrous tissue interfaced these areas [52].

### 3.5. SCAPs

SCAPs were isolated and characterized by Sonoyama et al. from immature roots of normal human impacted third molars [11].

They express mesenchymal stem cell markers, embryonic stem cell markers, and also neurogenic markers [11]. Unlike DPSCs and other MSCs, SCAPs are telomerase-positive, a characteristic of embryonic stem cells, which suggests a notably immature state of differentiation [25, 94]. SCAPs are able to differentiate into multiple mesenchymal lineages (osteoblasts, odontoblasts, adipocytes, chondrocytes, and smooth muscle cells) and neural lineage in vitro [25, 72, 95] and have higher proliferation ratio and mineralization ability than DPSCs whereas the adipogenic potential of SCAPs is weaker than BMMSCs [11, 95]. Similarly to other dental stem cells, SCAPs have been reported to have immunomodulatory characteristics [96].

Human SCAPs were transplanted into immunocompromised mice using particles of HA/TCP as a carrier and generated a typical dentin structure. In the same study both human SCAPs and PDLSCs have been transplanted in a minipig model to generate a root/periodontal complex capable of mimicking a biophysiological root/periodontal setup in vivo [25]. Another in vivo study reported that human SCAPs mixed with porous ceramic discs show hard tissue-forming potential transplanted into immunocompromised rats [55]. The human SCAPs' ability of generating bone tissues has been reported in a study in which they were transplanted into immunodeficient mice with a HA scaffold [72].

## 4. Conclusion

Dental stem cells are an easily obtainable source of multipotent cells and in vivo studies on animal models confirmed the significative outcomes of in vitro studies. Our review reports encouraging results concerning the scientific research on dental stem cells, particularly regarding their possible employment, together with scaffolds and GFs, as therapeutic tool in various branches of dentistry.

According to their differentiation capacity, every oral stem cell type represents a determined source for a specific application field (Table 2). The highest number of articles on this topic focuses on DPSCs, which are good candidates in regenerative endodontics for pulp organ regeneration into necrotic or vital but diseased teeth as well as for the induction of dentin tissue repair among exposed pulp. Even more recently discovered cells as SCAP and SHED can be suggested for use in regenerative endodontics. DPSCs, SHED, SCAPs, PDLSCs, and DFSCs are good candidates for improving the existing regenerative procedures of craniofacial bone defects together with already reliable scaffolds and/or GFs. PDLSCs and DFSCs can be proposed as adjuvants tools for periodontal regeneration procedures as GTR technique. Furthermore dental stem cells may provide innovative solutions also in other medical branches thanks to their multipotent differentiation ability and immunomodulatory properties.

Despite all, at present, there are no in vivo studies on humans supporting the reliability for therapeutic use and further evidence is required to demonstrate the possibility of using dental stem cells as a therapeutic tool for daily clinical practice.

## Figures and Tables

**Table 1:** In vivo studies dealing with dental stem cells applications in tissue engineering procedures.

Type of cell	Origin	Year	Reference	Scaffold	GFs	Animal model	Type of differentiation
DFSC	Bovine	2002	Handa et al. [52]	HA ceramic powder		Mice	Cementoblastic
	Human	2011	Honda et al. [53]	Pellet culture system		Rat	Osteoblastic
	Pig	2008	Tsuchiya et al. [54]	*β*-TCP		Mice	Osteoblastic
	Human	2005	Morsczeck et al. [10]	HA ceramic powder		Mice	Osteoblastic
	Human	2010	Yagyuu et al. [55]	Porous ceramic discs		Rat	Cementoblastic

DPSC	Dog, pig	2004	Iohara et al. [41]	Pellet culture system	BMP-2	Dog	Odontoblastic
	Human	2011	Alsanea et al. [37]	Collagen/dentin wafer	DMP-1	Mice	Odontoblastic
	Pig	2008	Prescott et al. [36]	Collagen/tooth slice	DMP-1	Mice	Odontoblastic
	Pig	2012	Kodonas et al. [44]	Collagen/PLGA		Pig	Odontoblastic
	Human	2011	Chan et al. [50]	Peptide hydrogel		Mice	Osteoblastic
	Human	2010	Demarco et al. [35]	PLLA/tooth slice and porogens		Mice	Odontoblastic
	Human	2000	Gronthos et al. [7]	HA/TCP ceramic powder		Mice	Odontoblastic
	Human	2010	Kraft et al. [51]	HA/TCP granules		Mice	Osteoblastic and odontoblastic
	Dog	2013	Wang et al. [45]	HA ceramic powder		Dog	Odontoblastic
	Dog	2013	Khorsand et al. [56]	Bovine bone granules		Dog	Osteoblastic, cementoblastic, and fibroblastic
	Rabbit	2008	El-Backly et al. [43]	DL-lactide-CO-glycolide		Rabbit	Odontoblastic
	Pig	2012	Zheng et al. [42]	*β*-TCP		Swine	Odontoblastic
	Rat	2013	Tsujigiwa et al. [46]	*β*-TCP particles		Mice	Odontoblastic
	Human	2014	Syed-Picard et al. [40]	3D scaffoldless DPSCs/root canal		Mice	Odontoblastic
	Human	2005	Laino et al. [48]	Fibrous bone obtained in vitro		Mice	Osteoblastic
	Human	2007	d'Aquino et al. [49]	Bone chips obtained in vitro		Mice	Osteoblastic

PDLSC	Dog	2012	Suaid et al. [57]	Collagen sponge		Dog	Cementoblastic and fibroblastic
	Dog	2012	Wang et al. [58]	HA ceramic particles		Mice	Fibroblastic
	Human	2004	Seo et al. [8]	HA/TCP ceramic particles		Rat	Cementoblastic and fibroblastic
	Dog	2009	Kim et al. [59]	HA/TCP granules		Dog	Osteoblastic
	Human	2011	Park et al. [60]	Macroporous biphasic calcium phosphate		Mice	Cementoblastic
	Human	2013	Ji et al. [61]	Human dentine bocks		Mice	Cementoblastic and fibroblastic
	Dog	2014	Yu et al. [62]	Bovine bone granules		Dog	Osteoblastic
	Rat	2014	Han et al. [63]	Adsorbable gelatin sponge		Rat	Osteoblastic, cementoblastic, and fibroblastic
	Human	2006	Sonoyama et al. [25]	HA/TCP blocks		Swine	Cementoblastic and fibroblastic

SHED	Human	2012	Wang et al. [64]	Ceramic bovine bone (CBB); fibrin gel		Mice	Osteoblastic
	Human	2010	Sakai et al. [65]	PLLA/tooth slice		Mice	Odontoblastic
	Human	2013	Rosa et al. [66]	Peptide hydrogel or rH collagen in root canal		Mice	Odontoblastic
	Human	2013	Alkaisi et al. [67]	Cell pellets		Rat	Osteoblastic
	Human	2014	Kim et al. [68]	Macroporous biphasic calcium phosphate	FGF-2	Mice	Odontoblastic
	Human	2010	Casagrande et al. [69]	PLLA/tooth slice		Mice	Odontoblastic
	Human	2008	Cordeiro et al. [70]	PLLA/tooth slice		Mice	Odontoblastic
	Human	2003	Miura et al. [9]	HA/TCP ceramic powder		Mice	Osteoblastic and odontoblastic
	Human	2008	Seo et al. [71]	HA/TCP particles		Mice	Osteoblastic

SCAP	Human	2006	Sonoyama et al. [25]	HA/TCP blocks		Swine	Odontoblastic
	Human	2012	Abe et al. [72]	HA/TCP particles		Mice	Osteoblastic
	Human	2010	Yagyuu et al. [55]	Porous ceramic discs		Rat	Odontoblastic

**Table 2:** The possible clinical application fields of dental stem cells and tissue engineering.

Cell type	Possible clinical application
DPSCs	(i) Regenerative endodontics (ii) Bone regeneration

SHED	(i) Regenerative endodontics (ii) Bone regeneration

SCAP	(i) Regenerative endodontics (ii) Bone regeneration

PDLSCs	(i) Periodontal regeneration (ii) Bone regeneration

DFSCs	(i) Periodontal regeneration (ii) Bone regeneration
